# Investigating ischemic vascular dysfunction in Alzheimer's disease

**DOI:** 10.1002/alz70855_105236

**Published:** 2025-12-24

**Authors:** Simone Woodruff, Bradley Marxmiller, Ozama Ismail, Martin Pike, Wenri Zhang, Randall Woltjer, Laura Villasana, Anusha Mishra

**Affiliations:** ^1^ Oregon Health & Science University, Portland, OR, USA; ^2^ Legacy Research Institute, Portland, OR, USA

## Abstract

**Background:**

Vascular dysfunction is one of the earliest pathological signs in the progression of Alzheimer's disease (AD). Ischemic injuries can cause long‐lasting vascular dysfunction in affected individuals and increase a patient's risk of developing dementia. Importantly, almost half of AD patients show indications of prior ischemia, emphasizing the importance of studying these conditions concurrently to better understand AD and related dementias. However, the mechanisms and factors that initiate and perpetuate vascular dysfunction in AD and related dementias are still unclear. We hypothesize that ischemic injury triggers widespread cerebrovascular dysfunction and accelerates the progression of AD.

**Method:**

We use an innovative mixed model of dementia, in which 6‐month‐old Tg2576 AD‐like model mice receive transient mild subcortical ischemia (tMSCI) via a 30‐minute middle cerebral artery occlusion. This is a particularly relevant model as subcortical strokes are the most common strokes in AD patients that correlate with dementia. We performed behavior assays to assess cognition, arterial spin labeling MRI and laser Doppler flowmetry to assess vascular function, and postmortem histology to examine astrogliosis and amyloid beta plaque pathology.

**Result:**

Our data demonstrate that even a mild ischemic event in adult AD and wildtype (WT) mice leads to chronic bilateral vascular dysfunction that persists with age. In addition, results indicate persistent reactive astrocytes after ischemic injury – astrogliosis is pronounced 8 months after tMSCI in both WT and Tg2576 mice. Finally, ischemia increased parenchymal amyloid beta plaque pathology, but not cerebral amyloid angiopathy, in Tg2576 mice.

**Conclusion:**

Our results reveal that mild ischemia leads to bilateral vascular dysfunction and persistent glial pathology at a chronic timepoint post‐ischemia. These pathologies are exacerbated and coincide with increased amyloid pathology in an AD mouse model. We are following this up with an ongoing cross‐sectional study to assess disease progression, including cognition, vascular function, and histopathology, at multiple timepoints after ischemia. Our goals are to understand how this cerebrovascular dysfunction initiates and progresses over lifespan and to correlate AD‐associated cognitive decline with pathophysiology. This project could reveal insights into the mechanism of ischemia‐induced chronic neurovascular impairments and how they contribute to the development of AD‐like dementia.

